# Bimodal nanobody agents for cancer imaging and potential intraoperative guidance: a systematic review

**DOI:** 10.1186/s12951-026-04192-w

**Published:** 2026-03-03

**Authors:** Najaf Mammadbayli, Betül Altunay, Quim Peña, Dmytro Kobzev, Agnieszka Morgenroth, Masoud Sadeghzadeh, Twan Lammers, Felix Manuel Mottaghy, Laura Schäfer, Susanne Lütje

**Affiliations:** 1 Department of Nuclear Medicine, University Hospital RWTH Aachen, Pauwelsstraße 30, Aachen, 52074 Germany; 2 Institute for Experimental Molecular Imaging, University Hospital RWTH Aachen, Aachen, 52074 Germany; 3 Center for Integrated Oncology (CIO), University Hospital RWTH Aachen, Aachen, 52074 Germany; 4 Department of Radiology and Nuclear Medicine, Maastricht University Medical Center, Maastricht, 6229 HX The Netherlands

**Keywords:** Single-domain antibody, Molecular imaging, Bimodal imaging tracer, Nanobody, Tumor targeting, Radiolabeled agent, Theranostics, Image-guided surgery

## Abstract

Bimodal imaging tracers that combine nuclear and optical modalities are gaining increasing relevance for in vivo applications in oncology, particularly for surgical guidance, where both real-time visualization and preoperative deep-tissue localization are crucial. Nanobodies are heavy-chain antibody fragments that offer unique advantages in this context, such as high specificity and rapid clearance, which allows for precise tumor localization and real-time surgical navigation. In this systematic review, we evaluate research studies reporting nanobody-based tracers for dual-modality imaging and analyze their design strategies, preclinical imaging performance, and translational progress. The analyses revealed that molecular targets commonly overexpressed in cancer cells, such as HER2, EGFR, and CEA have been the primary focus in the design of these tracers, together with widely used fluorophores like Cy5 and IRDye800CW combined with radionuclides such as gallium-68, technetium-99m, and copper-64. The preclinical performance of the reported tracers was highly promising, both in absolute tumor uptake and ability to achieve high-contrast images rapidly, as highlighted by a CD38-targeting tracer that produced a ~ 96-fold tumor-to-muscle ratio within hours of injection. While achieving stable and site-specific dual labeling remains a technical challenge, the combination of high target specificity and rapid background clearance makes nanobody-based systems particularly well-suited for generating high-contrast images on the same day of administration. This positions nanobodies as a versatile platform to develop tracers that enhance real-time image-guided surgery in oncology and ultimately improve patient outcomes.

**Graphical Abstract:**

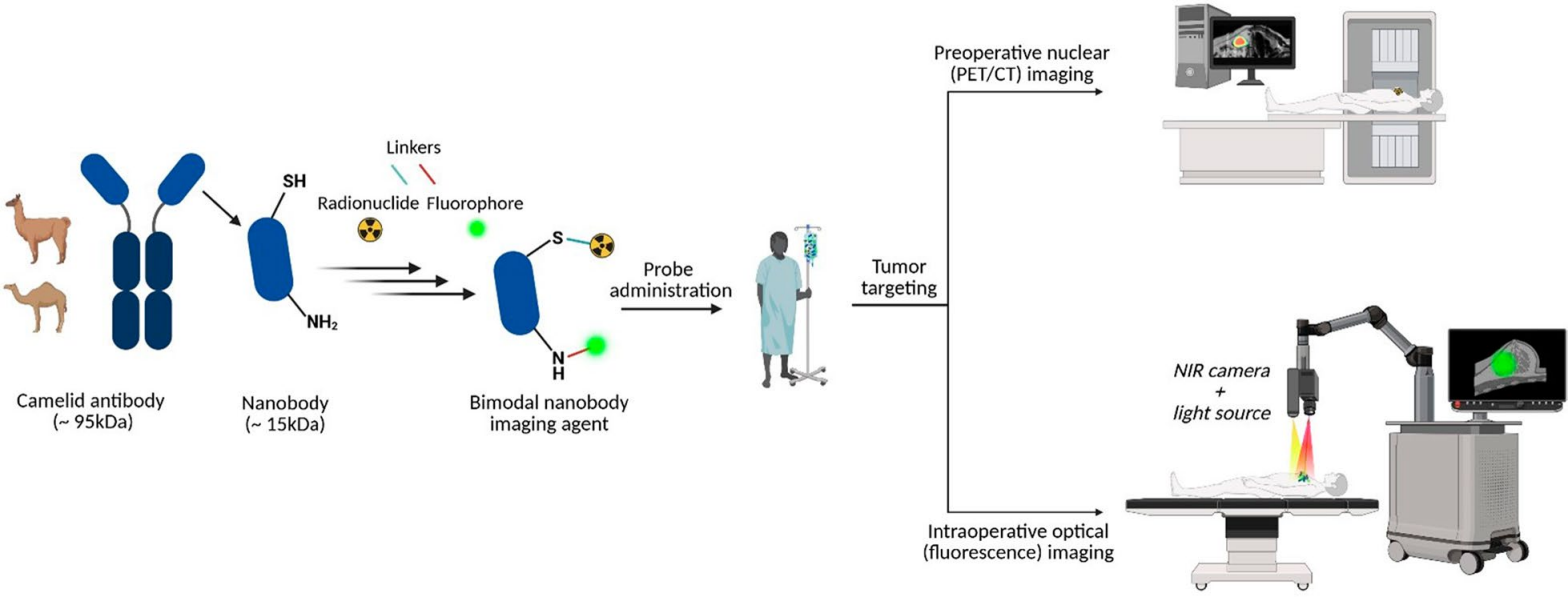

**Supplementary Information:**

The online version contains supplementary material available at 10.1186/s12951-026-04192-w.

## Introduction

In modern oncology, conventional anatomical imaging modalities like computed tomography (CT) are essential for visualizing the size and morphology of tumors [1, 2]. However, their anatomical focus limits the ability to provide the molecular-level information required for personalized cancer treatment, such as pre-selecting patients for targeted therapies or assessing treatment response [2]. To address these needs, the field of molecular imaging has developed targeted tracers, which typically consist of a signaling moiety conjugated to a targeting molecule [3]. In this construct, the targeting molecule guides the tracer to its specific biological target, while the signaling moiety generates the signal detected by the imaging scanner [3].

The signaling moiety determines the imaging modality and its capabilities [4]. As summarized in Table 1, distinct imaging modalities differ significantly in their physical principles, each presenting unique advantages and disadvantages regarding sensitivity, resolution, and clinical applicability. Radiolabeled agents called radiotracers, which use radionuclides as their signaling label, are employed for nuclear imaging techniques like positron emission tomography (PET) and single-photon emission computed tomography (SPECT) [5, 6]. The deep tissue penetration of the emitted radiation enables minimally invasive, whole-body tumor localization and quantitative assessment, although this is offset by a relatively low spatial resolution (typically several millimeters) [7, 8]. In contrast, fluorescence imaging relies on fluorescent molecules (fluorophores) as the signaling label instead of radionuclides [9]. These molecules do not emit ionizing radiation but provide a visual signal that can be detected in real-time, making them ideal for intraoperative guidance, but their clinical utility is constrained by the limited tissue penetration of light (1–2 cm) [2, 10]. Bimodal imaging seeks to overcome these inherent limitations by integrating both modalities, thereby synergizing the deep-tissue sensitivity of nuclear imaging with the real-time guidance of fluorescence imaging. Collectively, in contrast to purely anatomical modalities, molecular imaging approaches offer high sensitivity and specificity [1, 2], with PET being able to detect radiolabeled agents at picomolar concentrations, and thus enabling the detection of tumors at an earlier stage, which facilitates tailored and timely therapeutic interventions [1, 5].

**Table 1:** Comparative overview of the characteristics, advantages, and limitations of standard clinical imaging modalities and fluorescence imaging in oncology

**Property**	**CT** [11, 12]	**SPECT** [8, 13]	**PET** [8, 11]	**Fluorescence** [14, 15]
Primary signal	X-ray	Gamma rays (*γ*)	Positrons (*β*^+^)/ *γ*-rays	Light excitation and emission
Tissue penetration	Deep (whole-body)	Deep (whole-body)	Deep (whole-body)	Limited (mm to cm range)
Spatial resolution	High (< 1 mm)	Low (~ 6–15 mm)	Moderate (~ 3–6 mm)	High (microns to mm)
Sensitivity	Low (millimolar)	High (nanomolar)	Very high (picomolar)	High (nanomolar)
Primary clinical role	Anatomical localization	Diagnosis and staging	Diagnosis, staging and quantification	Intraoperative guidance
Key Advantages	High anatomical detail, Rapid acquisition	Simultaneous multi-isotope imaging, Widely available	High sensitivity, Quantitative capability	Real-time visualization, High resolution, Non-radioactive
Key Limitations	No molecular information, Radiation exposure, Low soft-tissue contrast	Radiation exposure, Lower sensitivity than PET, Long acquisition times	Radiation exposure, Low spatial resolution, High cost	Low depth penetration, High signal attenuation, Qualitative only

Equally important is the choice of the targeting molecule to provide specificity and adequate image contrast, while its size and physicochemical properties determine the tracer’s pharmacokinetic profile [4]. The main classes of currently used targeting molecules range from small molecules (e.g. PSMA [16] or FAPI [17] ligands, with molecular weight (MW) < 2 KDa [18] to larger macromolecules. These macromolecules include large monoclonal antibodies (~ 150 kDa) [19, 20], small peptides (< 5 kDa) [21, 22], and, more recently, nanobodies, which are small heavy-chain antibody fragments (single-domain antibodies) of about 15 kDa [23, 24], each offering distinct advantages and limitations (Table 2). Antibodies, for example, exhibit long circulation times, leading to high tumor accumulation but requiring multi-day imaging protocols [20]. In contrast, small peptides are often cleared too rapidly, which allows for minimal background signal but can result in insufficient tumor uptake [21]. Nanobodies, with their intermediate size, offer an optimal balance between effective tumor uptake and rapid background clearance, making them particularly suitable for applications requiring same-day, high-contrast images, such as image-guided surgery [24]. These single-domain antibodies are the smallest antigen-binding fragments, originating from the heavy-chain-only antibodies found in camelids [25, 26]. In addition to their rapid renal clearance, a unique combination of other properties including good water-solubility, high thermal and chemical stability, generally low immunogenicity, and scalable production in microbial systems has increased the interest and potential of nanobodies for molecular imaging [26, 27].

**Table 2:** Comparative properties of common targeting agents for molecular imaging

**Property**	**Peptides** [28]	**Nanobodies** [23]	**Antibodies** [19]
Size/ Molecular weight	Very small (< 5 kDa)	Small (~ 15 kDa)	Large (~ 150 kDa)
Blood half-life	Very short (minutes)	Short (minutes to hours)	Very long (days to weeks)
Tumor penetration	Very rapid	Rapid	Slow
Optimal imaging time	Very early (< 1 h)	Early (1–4 h)	Delayed (3–7 days)
Primary clearance route	Renal (kidney)	Renal (kidney)	RES (liver and spleen)
Key advantage	Fast background clearance	Optimal balance for same-day imaging	High absolute tumor uptake
Key limitation	Potentially low tumor retention	High renal uptake	Slow bloodstream clearance for same-day surgery

Despite the abovementioned features, a single modality is often insufficient to bridge the gap between preoperative planning and real-time surgical guidance, making the combination of different modalities, such as nuclear and optical, highly advantageous [29]. Preoperatively, the deep-tissue sensitivity of nuclear imaging allows for whole-body scans to map the extent of the disease [4]. Intraoperatively, this is complemented by real-time feedback where a surgeon can first use a gamma probe to acoustically locate deeper lesions and then, upon surgical exposure, use the visual fluorescent signal for precise margin delineation [30]. This combination is particularly valuable in complex clinical scenarios such as head and neck cancer surgery, where accurate definition of deep tumor margins using PET or SPECT is critical [31, 32], and real-time fluorescence imaging helps avoid damage to nearby nerves and vessels [10].

To address the limitations of single-modality imaging, bimodal imaging tracers integrating nuclear and fluorescent modalities into a single platform have shown promise [9]. These tracers offer a more complete surgical solution by combining the deep tissue sensitivity of nuclear imaging for preoperative planning with the real-time visual feedback of fluorescence for precise intraoperative guidance [33].

Nanobodies are well-suited candidates for bimodal imaging agents due to their stability and inherent modularity, providing multiple conjugation strategies (e.g., non-site specific labeling of surface lysines or site-specific attachment via engineered cysteines) for attaching various imaging payloads [34] while retaining full biological activity throughout the entire labeling process [35, 36]. This enables the development of multifunctional tracers for both real-time visualization at the surgical site and systemic tumor localization.

While previous reviews have focused on either general nanobody radiolabeling or broad bimodal imaging strategies, this review aims to systematically evaluate the preclinical and clinical landscape of nanobody-based tracers utilizing nuclear and optical labeling strategies. We evaluate both true bimodal agents intended for clinical translation and those utilizing independent labeling approaches for preclinical validation. Specifically, we provide a critical analysis of the various chemical conjugation strategies that have been employed to address the complexities associated with dual functionalization and the preclinical performance data to highlight both successful imaging outcomes and key biological challenges, such as off-target uptake in kidneys. The overall goal is to provide a comprehensive summary of the current state-of-the-art and to identify the next critical steps needed to advance nanobody-based theranostic bimodal tracers toward translation and clinical application.

## Methods

### Search strategy

This systematic review was conducted in line with the PRISMA (Preferred Reporting Items for Systematic Reviews and Meta-Analyses) guidelines [37]. To ensure consistent terminology, this review uses the following definitions: the general term “tracer” describes all targeted nanobody-based constructs; “radiolabeled agent” refers specifically to those for nuclear imaging; and “fluorescent probe” refers to those for optical imaging. To identify relevant studies on bimodal nanobody-based imaging tracers – particularly those involving both a fluorescent dye and a radiolabel – a structured search of the **PubMed, Web of Science,** and **ClinicalTrials.gov** databases was carried out. The literature search covered articles published from database inception up to June 2025, using the search terms “intraoperative nanobody”, “multimodal nanobody”, “real time imaging nanobody”, “nanobody AND fluorescence AND radionuclide” and “nanobody AND imaging AND (PET/CT OR SPECT)”. The aim of the search was to identify all relevant studies and articles involving nanobody-based imaging tracers labeled with more than one modality (e.g., nuclear and optical), as well as related approaches using separately conjugated agents and probes or nanoparticle platforms. Only original research articles published in peer-reviewed journals and written in English were included.

### Inclusion and exclusion criteria

#### Inclusion criteria

Studies were included if they met **all** the following criteria:

- Bimodal Nanobody Labeling: The study focused on nanobodies, or nanobody-conjugates, that were labeled with both a fluorescent dye and a radionuclide – either together on the same construct or through separate experiments using the same nanobody.
- Original Experimental Data: The study included original experimental results from in vitro experiments, in vivo*, * animal studies, or human clinical trials.
- Peer-Reviewed Full Text: The article was published in a peer-reviewed scientific journal, with full text available in English and featured detailed, accessible methodology and results.

#### Exclusion criteria

Studies were excluded based on **any** of the following criteria:

- No bimodal labeling: Studies that involved only fluorescence or only radiolabeling without combining both modalities in the same nanobody system.
- Non-original or Incomplete Reports: Review papers, book chapters, editorials, or conference abstracts without full original data.
- Different Nanobodies: The study used different nanobodies for fluorescence and radiolabeling, rather than dual labeling a single nanobody with both modalities.
- Duplicate Publications: Identical records identified from overlapping database searches, or duplicate reports of the same study published in more than one article, with preference given to the most complete version.

### Study selection

All articles retrieved from the literature search were exported into Microsoft Excel, where duplicates were manually detected and removed. Following that, the entire screening and selection process, including the initial title and abstract review and the final full-text eligibility assessment, was manually performed independently by two authors (NM and LS), without the use of automated tools, with any disagreement resolved by discussion to reach the final consensus. A PRISMA flow diagram was generated to visually summarize the selection process, including the number of records identified, screened, included, and excluded in the review, as well as the reasons for the exclusion at each stage.

### Data synthesis

Due to the heterogeneity among the included studies in terms of imaging modalities, molecular targets, tracer design, and functionalization strategies, as well as the predominantly preclinical nature of the data, a quantitative meta-analysis was not possible. The collected data were categorized thematically based on the following aspects:

- Imaging Modalities: Classifying nanobody-based tracers by imaging modality and integration of nuclear and optical labels, either in one construct or separately.
- Bioconjugation Strategies for Bimodal Tracer Development: Grouping tracer design approaches into direct dual labeling, scaffold-based conjugation, and independent labeling strategies.
- Technical Challenges in Tracer Synthesis and Functionalization: Outlining challenges in dual functionalization, from multi-step labeling to nanobody-specific challenges and off-target signal.
- Preclinical Evaluation and Imaging Outcomes: Summarizing tracer performance in animal models, focusing on tumor targeting, imaging contrast, biodistribution, and clearance behavior.
- Translation to Clinical Settings: Reviewing early clinical findings, highlighting patient imaging, tracer safety, and prospective intraoperative applications.

This structure enabled comparison of nanobody-based tracer designs, imaging outcomes, and gaps in clinical translation.

### Risk of bias analysis

A risk of bias assessment was conducted for the 14 in vivo studies (12 preclinical and 2 clinical) included in this review (Tables 3A and 3B). The SYRCLE tool [38] was applied to the 12 preclinical animal studies, while the QUADAS-2 tool [39] was used for the two clinical trials. The remaining single study that reported only in vitro data was excluded from this analysis, as these tools are not applicable to its design.

**Table 3A:** Summary of risk of bias analysis. Part A: Risk of bias for preclinical animal studies

SYRCLE domain	[40]	[41]	[42]	[43]	[44]	[45]	[46]	[47]	[48]	[49]	[50]	[51]
Sequence generation	Unclear	Low	Unclear	Unclear	Unclear	Unclear	Unclear	Unclear	Unclear	Unclear	Unclear	Unclear
Baseline characteristics	Unclear	Low	Low	Unclear	Unclear	Unclear	Unclear	Unclear	Unclear	Low	Unclear	Unclear
Allocation concealment	Unclear	Low	Unclear	Unclear	Unclear	Unclear	Unclear	Unclear	Unclear	Unclear	Unclear	Unclear
Random housing	Unclear	Unclear	Unclear	Unclear	Unclear	Unclear	Unclear	Unclear	Unclear	Unclear	Unclear	Unclear
Blinding of researchers	Unclear	Unclear	Unclear	Unclear	Unclear	Unclear	Unclear	Unclear	Unclear	Unclear	Unclear	Unclear
Random outcome assessment	Unclear	Unclear	Unclear	Unclear	Unclear	Unclear	Unclear	Unclear	Unclear	Unclear	Unclear	Unclear
Blinded outcome assessment	Unclear	Unclear	Unclear	Unclear	Unclear	Unclear	Unclear	Unclear	Unclear	Unclear	Unclear	Unclear
Incomplete outcome data	Low	Low	Low	Unclear	Low	Low	Low	Low	Low	Low	Low	Low
Selective outcome reporting	Low	Low	Low	Low	Low	Low	Low	Low	Low	Low	Low	Low
Other sources of bias	Unclear	Unclear	Unclear	Unclear	Unclear	Low	Unclear	Unclear	Unclear	Low	Unclear	Unclear

**Table 3B:** Summary of risk of bias analysis. Part B: Risk of bias for clinical diagnostic studies

QUADAS-2 domain	[52]	[53]
Patient selection	Unclear	Unclear
Index test	Unclear	Unclear
Reference standard	Unclear	Unclear
Flow and timing	Unclear	Low

For the preclinical studies, the overall methodological quality was difficult to ascertain due to insufficient reporting across most domains (Table 3A). While the majority of studies were judged to be at a low risk of bias for incomplete outcome data and selective reporting, a high degree of unclear risk was identified for domains related to selection, performance, and detection bias. Specifically, most studies lacked details on sequence generation, allocation concealment, and blinding. A similar pattern was also observed for the two clinical trials (Table 3B). The risk of bias concerning patient selection, the index test, and the reference standard was rated as unclear for both studies, primarily because key information on the enrollment process and blinding procedures was missing. The domain of flow and timing was judged to be at a low risk of bias for one of the two trials. The complete, itemized risk of bias assessment for each of the 14 included studies is provided in Additional file 1.

## Results

### Literature selection

**Fig. 1:**
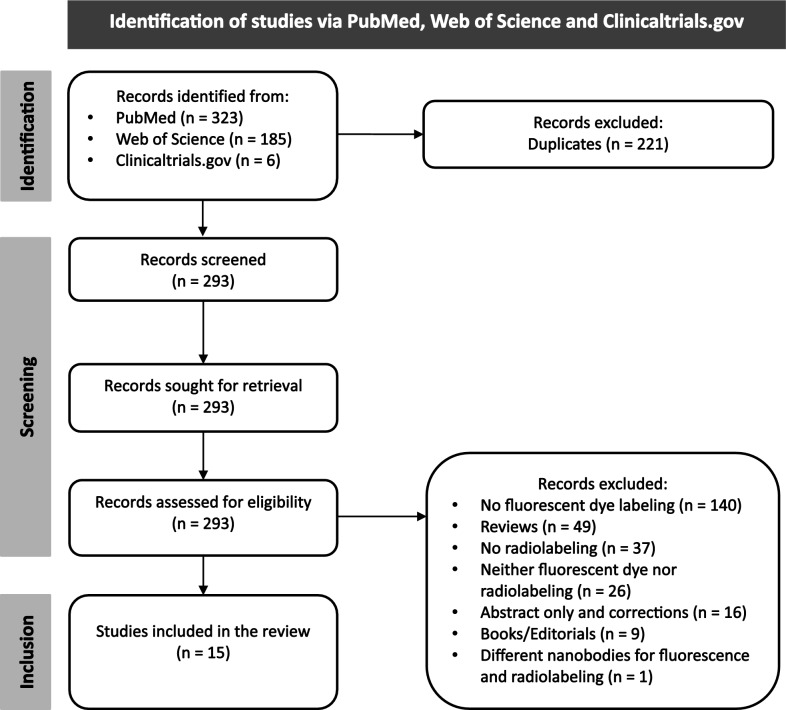
Flowchart of the selection process for the literature included in this study. From an initial 514 records, 221 duplicates were removed. The remaining 293 articles were screened, resulting in 15 studies meeting the inclusion criteria for the final analysis

The comprehensive literature search across PubMed, Web of Science, and ClinicalTrials.gov yielded 514 records, with the detailed breakdown of search terms presented in Table 4. After the removal of 221 duplicates, the remaining 293 records were screened. Following a full-text assessment, 15 peer-reviewed articles (14 reporting in vivo data) met the eligibility criteria and were included for critical analysis, as illustrated in Fig. 1.

**Table 4:** Number of records retrieved from PubMed, Web of Science, and Clinicaltrials.gov based on various search terms related to bimodal nanobodies in imaging

Keywords	Database	Number of literature results
“Intraoperative nanobody”	PubMed	12
	Web of Science	9
	Clinicaltrials.gov	0
“Multimodal nanobody”	PubMed	89
	Web of Science	19
	Clinicaltrials.gov	0
“Real time imaging nanobody”	PubMed	35
	Web of Science	22
	Clinicaltrials.gov	0
“Nanobody AND imaging AND (PET/CT OR SPECT)”	PubMed	173
	Web of Science	133
	Clinicaltrials.gov	6
“Nanobody AND radionuclide AND fluorescence”	PubMed	14
	Web of Science	2
	Clinicaltrials.gov	0

### Imaging modalities

**Fig. 2:**
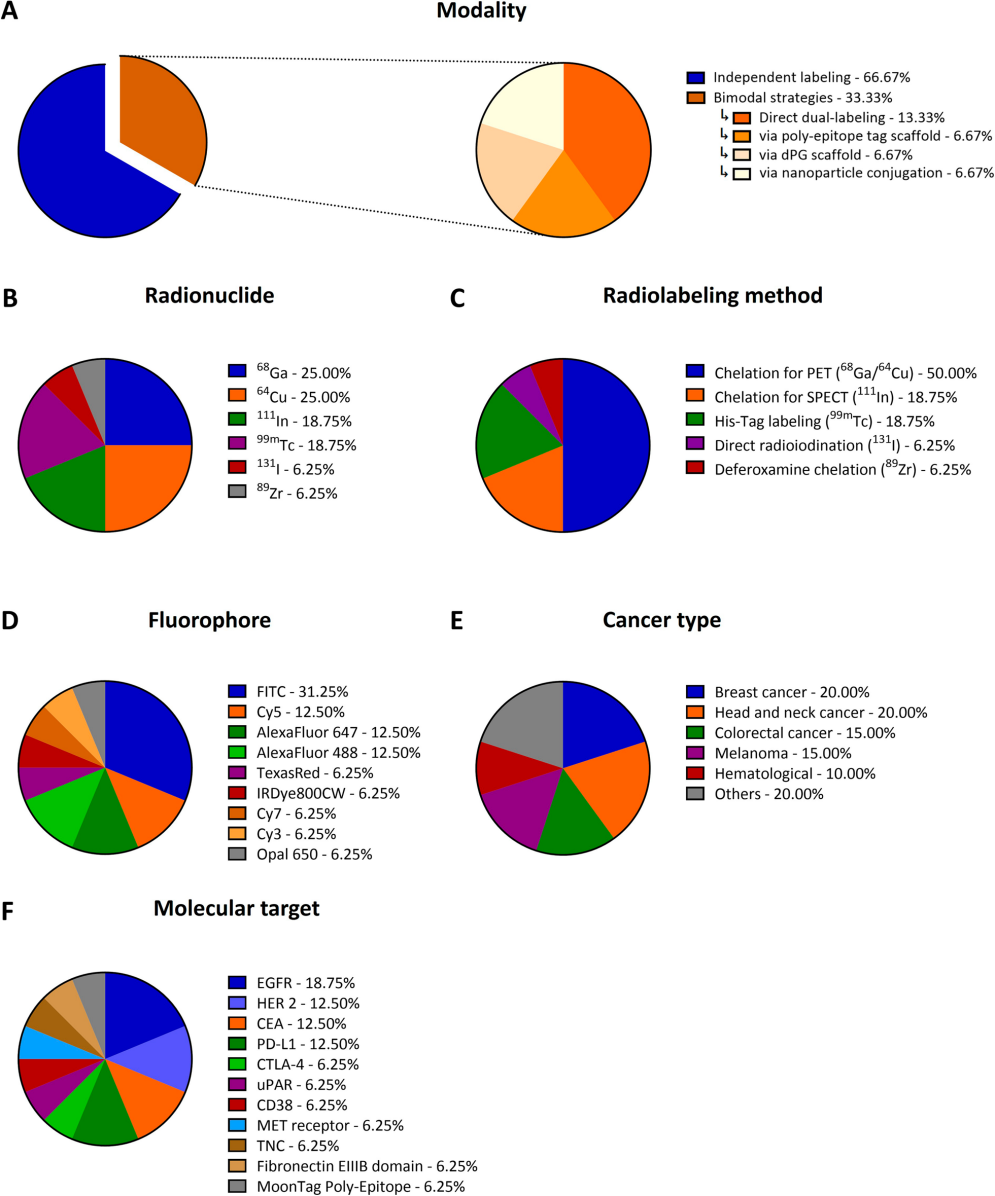
Schematic distribution of key parameters from reviewed studies. The charts show the distribution of **A)** modality, **B)** radionuclide, **C)** radiolabeling method, **D)** fluorophore type, **E)** cancer type and **F)** molecular target. The total number of entries per chart can be higher than the total number of included studies (n = 15) since some papers reported multiple parameters within a single category. Abbreviations: dPG (dendritic polyglycerol); EGFR (epidermal growth factor receptor); HER2 (human epidermal growth factor receptor 2); CEA (carcinoembryonic antigen); PD-L1 (programmed death-ligand 1); CTLA-4 (cytotoxic T-lymphocyte–associated protein 4); CD38 (cluster of differentiation 38); MET (receptor tyrosine kinase MET); TNC (tenascin C); uPAR (urokinase plasminogen activator receptor)

A key distinction among the 15 included studies was the coupling strategy used to combine nuclear and optical imaging (Fig. 2A). Most of the studies (n = 10) focused on independently labeled nanobodies, where the same nanobody was separately functionalized with either a radionuclide or a fluorophore for comparative analysis. The remaining five studies successfully developed bimodal tracers, where both imaging labels were directly integrated in a single nanobody construct.

For nuclear imaging, different radionuclides for both PET and SPECT were utilized (Fig. 2B). Among the included studies, PET was more frequently reported, with gallium-68 (^68^Ga) [42, 44, 52, 53] and copper-64 (^64^Cu) [43, 46, 51, 54] being the most explored isotopes, each being used in four of the studies. For SPECT applications, technetium-99m (^99m^Tc) [41, 48, 49] and indium-111 (^111^In) [40, 44, 50] were each employed in three studies. In contrast, the long-lived PET-radionuclide zirconium-89 (^89^Zr) [47] and the theranostic agent iodine-131 (^131^I) [45] were used less frequently, each featured in one study.

For optical imaging, a wide variety of fluorophores were utilized, ranging from the visible to the near-infrared (NIR) spectrum (Fig. 2D), a distinction that dictates their tissue penetration depth. The most frequently reported dyes were in the visible range. These included fluorescein isothiocyanate (FITC; λ_ex_ = 494 nm/ λ_em_ = 518 nm), which appeared in five studies [45, 48, 52–54], followed by Alexa Fluor 488 (λ_ex_ = 495 nm/ λ_em_ = 519 nm), utilized in two studies [42, 49]. Other visible dyes like Texas Red [46] (λ_ex_ = 595 nm/ λ_em_ = 613 nm) and Cy3 [43] (λ_ex_ = 550 nm/ λ_em_ = 570 nm) were used less frequently, each appearing in a single study. While these dyes are standard for ex vivo validation (e.g., flow cytometry or immunofluorescence), their emission wavelengths fall outside the biological optical window, limiting their utility for deep-tissue in vivo imaging due to the high photon scattering and absorption.

In contrast, tracers designed for in vivo applications employed far-red and NIR fluorophores. The most prominent ones were the hydrophilic, sulfonated cyanine dyes Cy5 (λ_ex_ = 649 nm/ λ_em_ = 670 nm) and Cy7 (λ_ex_ = 743 nm/ λ_em_ = 767 nm), which together were reported across three different studies [40, 43, 44] and Alexa Fluor 647 (λ_ex_ = 650 nm/ λ_em_ = 668 nm), appearing in two of the studies [50, 51]. Additionally, the far-red dye Opal 650 [47] (λ_ex_ = 627 nm/ λ_em_ = 650 nm) and the specific NIR dye IRDye800CW [41] (λ_ex_ = 774 nm/ λ_em_ = 789 nm) were each reported once. These fluorophores emit light within the “biological optical window” (650–900 nm), significantly reducing tissue autofluorescence compared to visible dyes [55]. However, the optimal depth of penetration is achieved primarily by the longer-wavelength NIR dyes (such as IRDye800CW and Cy7), making them the preferred choice for clinical intraoperative navigation in deep tissues. A comprehensive overview of the radionuclides, fluorophores, and imaging modalities used in each study is provided in Table 5.

**Table 5:** Summary of multimodal nanobody tracers in cancer imaging

Modality	Lead compound	Fluorophore	Radiolabeling method	Molecular target	Tumor type	Clinical application	Ref
Bimodal	2Rs15d	Cy5	^**111**^**In** with DTPA	HER2	Ovarian cancer	-	[40]
	uPAR15-GEM	IRDye800CW	^**99m**^**Tc** via His_6_ -Tag	uPAR	Colorectal cancer	-	[41]
Bimodal (via poly-epitope tagging)	MoonTag Nb	Alexa Fluor 488	^**68**^**Ga** with NODAGA	MoonTag Poly-Epitope	Ewing sarcoma	-	[42]
Bimodal (via dPG scaffold)	α-EGFR-dPG	Cy3/Cy7	^**64**^**Cu** with DMPTACN	EGFR	Epidermoid carcinoma	-	[43]
Bimodal (via NP-conjugation)	sdAb 7C12-Si-NPs	FITC	^**64**^**Cu** with NOTA	EGFR	Hypopharyngeal cancer	-	[54]
Independently labeled	2Rs15d	Cy5	^**111**^**In** with CHX-A’’-DTPA / ^**68**^**Ga** with NOTA	HER2	Breast cancer	-	[44]
	HNI01	FITC	^**68**^**Ga** with THP	CEA	Primary and metastatic colorectal carcinoma	Phase I clinical trial	[52]
	KN046	FITC	^**131**^**I** direct labeling	PD-L1 and CTLA-4	Malignant melanoma	-	[45]
	NJB2	Texas Red	^**64**^**Cu** with NOTA	Fibronectin EIIIB (EDB) domain	Triple negative breast cancer, pancreatic ductal adenocarcinoma, melanoma	-	[46]
	1E7-Fc	Opal 650	^**89**^**Zr** with p-SCN-Bn-Deferoxamine	MET receptor	Head and neck squamous cell carcinoma	Clinical tissue analysis	[47]
	CD3813	FITC	^**68**^**Ga** with TOHP	CD38	Multiple myeloma and CD38 + lymphomas	Phase I clinical trial (NCT06385652)	[53]
	15.2 m	FITC	^**99m**^**Tc** via His_6_ -Tag	CEA	Non-small cell lung cancer	-	[48]
	D10	Alexa Fluor 488	^**99m**^**Tc** via His_6_ -Tag	EGFR	Breast cancer and epidermoid carcinoma	-	[49]
	K2	Alexa Fluor 647	^**111**^**In** with DOTA	PD-L1	Melanoma	-	[50]
	NJT6	Alexa Fluor 647	^**64**^**Cu** with NOTA	TNC	Triple negative breast cancer, colorectal cancer	-	[51]

### Bioconjugation strategies for bimodal tracer development

**Fig. 3:**
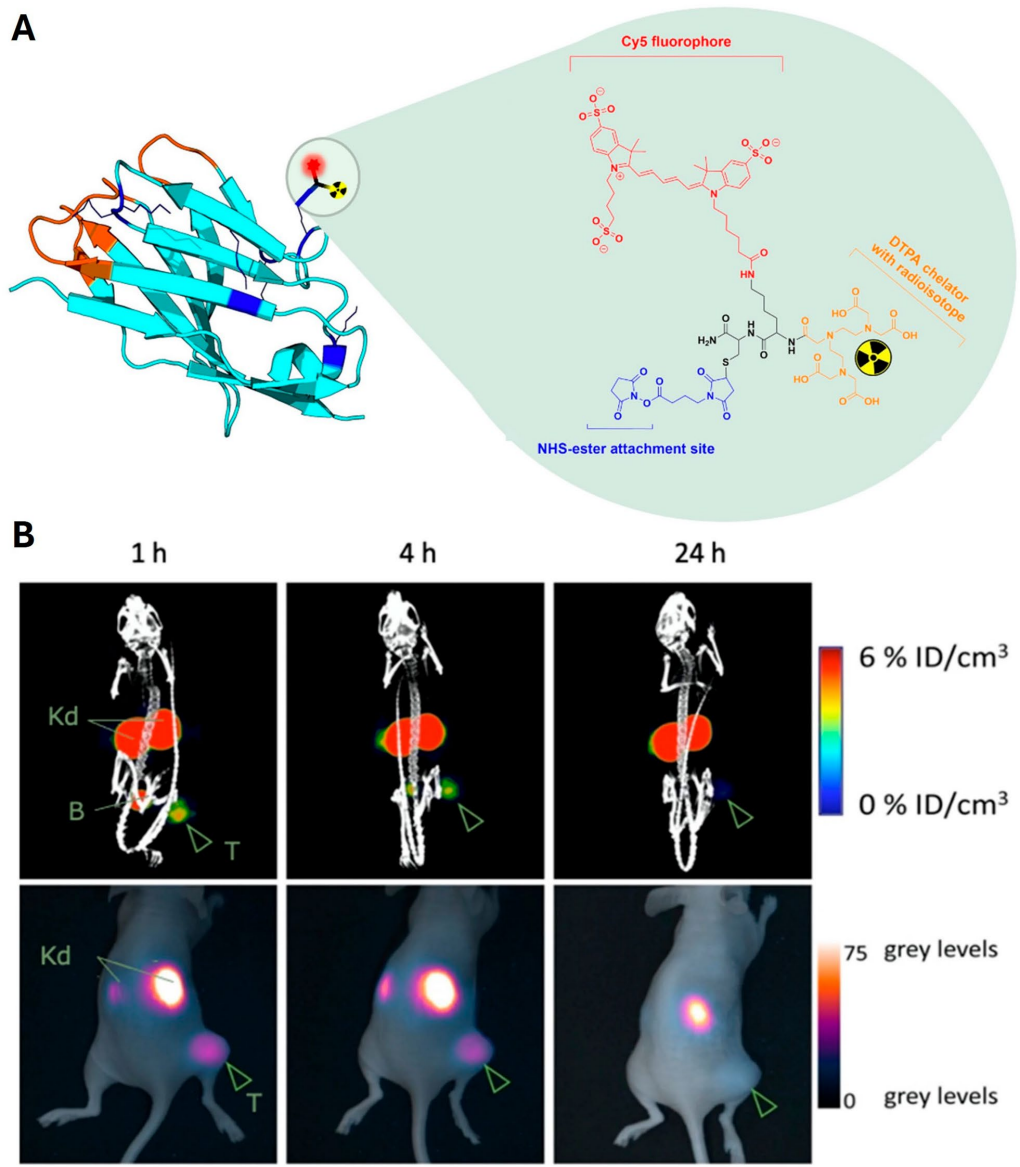
Concept and preclinical performance of bimodal nanobody-imaging agent [^111^In]In-MSAP.2Rs15d. **A)** Schematic representation of the anti-HER2 nanobody 2Rs15d being randomly labeled on the lysines via *NHS*-activated ester bioconjugation chemistry (blue) with the MSAP (multifunctional single attachment point) containing a Cy5 dye (red) and a DTPA-chelator complexed with an indium-111 (^111^In) radioisotope (yellow). The MSAP analogue’s backbone is displayed in black. **B)** Representative SPECT/CT (top) and fluorescence (bottom) images of an ovarian (SKOV3) tumor-bearing mouse at 1 h, 4 h, and 24 h post-injection of [^111^In]In-MSAP.2Rs15d. Specific uptake can be observed in the tumor (T), with little to no non-specific uptake, except for in the kidneys (Kd) and the bladder (B). This results in the specific and high-contrast imaging of HER2-positive subcutaneous tumors. These images were adapted from Debie P, Declerck NB, van Willigen D, Huygen CM, De Sloovere B, Mateusiak L, et al. The Design and Preclinical Evaluation of a Single-Label Bimodal Nanobody Tracer for Image-Guided Surgery. Biomolecules. 2021;11(3):360

The majority of the reviewed publications utilized independently labeled nanobodies to validate each imaging tracer separately. Notably, none of these studies reported the co-administration of the two tracers to confirm their in vivo co-localization. The remaining five studies that developed true bimodal tracers employed several strategies for bioconjugation of the imaging agents to the nanobody (Fig. 2A). These approaches can be broadly categorized into two main functionalization methods: direct dual labeling and scaffold-mediated conjugation. In the direct labeling strategy, both the fluorophore and the chelator were individually attached to the nanobody backbone, representing the most straightforward method of generating a bimodal construct. This was applied in two studies with distinct chemical approaches: a tracer targeting human epidermal growth factor receptor 2 (HER2) was functionalized via non-site-specific conjugation, using *N*-hydroxysuccinimide ester-activated chelators and dyes to target surface lysine residues of the nanobody (Fig. 3A) [40], while another targeting urokinase plasminogen activator receptor (uPAR) employed a dual site-specific approach, using a C-terminal hexahistidine-tag for radiolabeling and a separate maleimide group for the fluorophore [41].

**Fig. 4:**
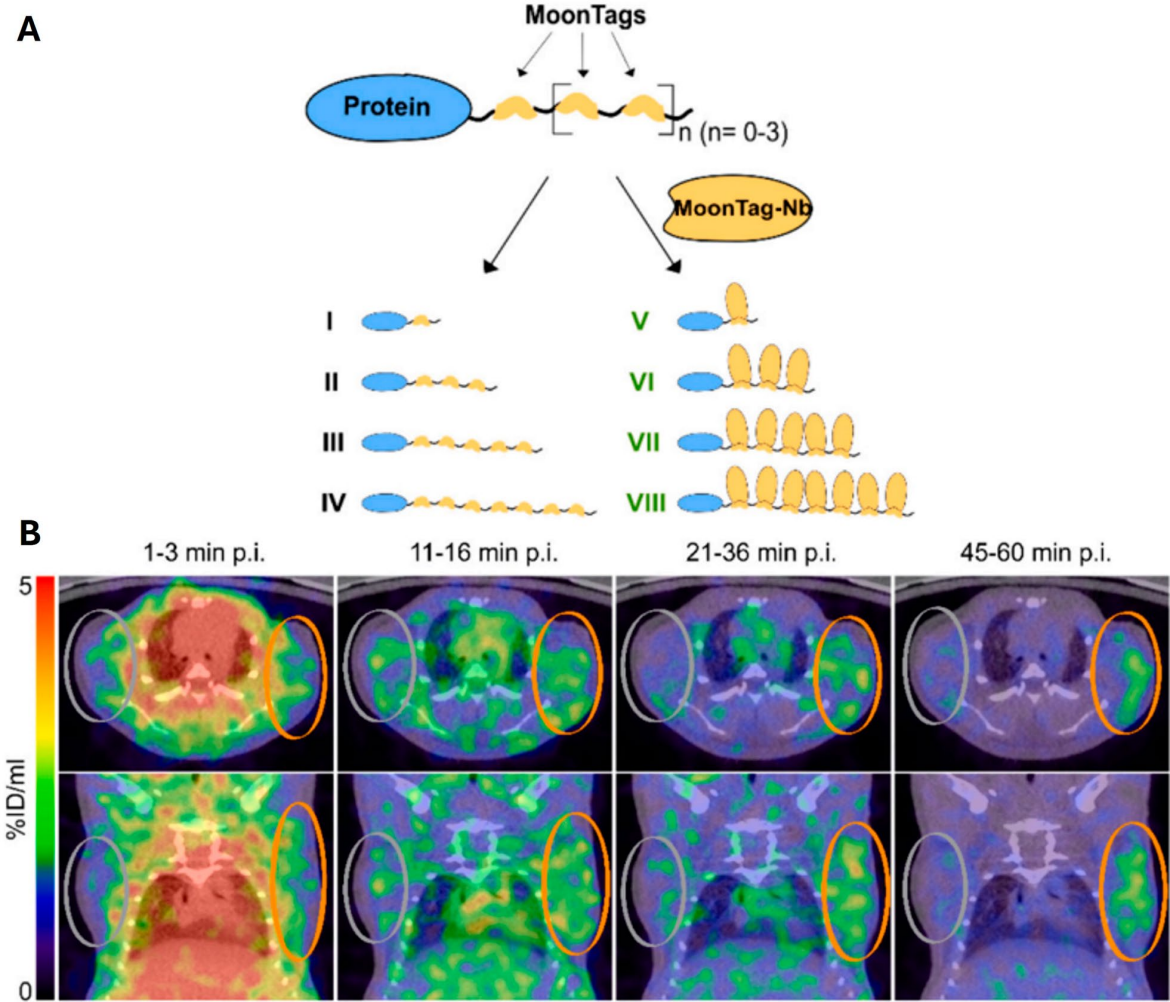
Concept and preclinical performance of the scaffold-mediated nanobody-imaging system [^68^Ga]Ga-NODAGA-MoonTag.** A)** Schematic representation of the modular MoonTag strategy. The target protein is fused to a peptide scaffold containing multiple MoonTag epitopes (orange), which serve as docking sites. These sites are subsequently bound by a radiolabeled "detector" nanobody (MoonTag-Nb), allowing for signal amplification and flexible modular labeling with fluorescence through the formation of a multi-epitope complex. **B)** Representative transaxial and coronal PET/CT images of tumor xenograft-bearing NSG mice after intravenous injection with [^68^Ga]Ga-NODAGA-MoonTag-Nb. Specific and high-contrast uptake is observed in the 7xMoonTag-expressing tumor xenografts (orange circle) over time, compared to the absence of signal in the wild-type control tumors (gray circle). This demonstrates the feasibility of high-contrast imaging using a scaffold-based assembly in vivo. These images were adapted from Höffgen KS, Dabel J, Konken CP, Depke DA, Hermann S, Dörner W, et al. Combining poly-epitope MoonTags and labeled nanobodies for signal amplification in cell-specific PET imaging in vivo. Nucl Med Biol. 2024;136–137:108,937

The other three studies utilized a more advanced scaffold-mediated conjugation strategy to achieve a more complex functionalization. One study developed a poly-epitope tag system for modular labeling (Fig. 4A) [42]; another utilized the branched structure of a dendritic polyglycerol (dPG) scaffold for co-labeling with multiple macrocyclic chelators and fluorescent dyes to target epidermal growth factor receptor (EGFR) [43]; and a third study used silicon nanoparticles as a large carrier platform, which was functionalized with targeting nanobodies via a thioether bond using maleimide chemistry [54].

These functionalization strategies offer distinct advantages and disadvantages, while also dictating the performance profile of the tracer. The direct dual-labeling method is chemically straightforward and maintains the nanobody’s small size (~ 15 kDa), favoring rapid tumor penetration and clearance (Fig. 3B) [40], although it carries the risk of steric hindrance and reduced target affinity due to attachment of bulky labels near the binding site [40, 41]. In comparison, scaffold-based approaches enable a higher payload of imaging agents for enhanced signal sensitivity as demonstrated by the MoonTag system (Fig. 4B) [42]. However, the increased molecular weight of these scaffolds [42, 43, 54] can alter pharmacokinetics by slowing blood clearance and potentially increasing the background noise.

Besides the nanobody bioconjugation approach, the choice of the radionuclide and radiolabeling strategy also represented a central aspect of the bimodal tracer development (Fig. 2C). Metal chelation was the most prevalent radiolabeling method, employed in all but one of the reviewed studies. The most common approach involved the use of macrocyclic chelators such as 1,4,7-triazacyclononane-1,4,7-triacetic acid (NOTA) for ^64^Cu(II) and ^68^Ga(III) [44, 46, 51, 54], or 1,4,7,10-tetraazacyclododecane-1,4,7,10-tetraacetic acid (DOTA) and diethylenetriaminepentaacetic acid (DTPA) for ^111^In(III) [40, 44, 50], which coordinate the metals using *N*- and/or *O*-donor atoms. In addition to these conventional methods, three studies employed the site-specific chelation of ^99m^Tc(I) by a hexahistidine-tag, offering greater control over the final construct [41, 48, 49], while another study used deferoxamine (DFO) chelator for the chelation of ^89^Zr(IV) [47]. The only non-chelation-based method reported was direct radioiodination with the non-metallic ^131^I isotope, where iodine is electrophilically attached to the tyrosine residues of the nanobody [45].

### Technical challenges in tracer synthesis and functionalization

The reviewed literature highlights several technical challenges associated with the synthesis and functionalization of these nanobody tracers, primarily related to controlling conjugation chemistry and achieving consistent labeling outcomes in terms of purity, radiochemical yield, and in vivo stability. A major issue across multiple studies was the difficulty in attaining molecular homogeneity during the labeling process. Conventional non-selective conjugation methods targeting lysine residues were reported to yield heterogeneous mixtures of products [40, 50]. For instance, this was specifically demonstrated for the programmed death-ligand 1 (PD-L1)-targeting K2 nanobody, which, after DOTA conjugation, resulted in a mixture of unconjugated and mono-conjugated forms [50]. To address this, various site-specific strategies like Sortase A-mediated conjugation [44] and cysteine-maleimide chemistry [41, 42, 49, 50, 52, 53] were employed to produce more homogeneous products. However, these methods can introduce additional complexities. A key example is cysteine-tagging, which was reported to reduce production yield and require controlled reduction steps to avoid side products [41].

Beyond conjugation control, the overall complexity of tracer synthesis emerged as a significant limiting factor, with one study describing the generation of a multi-labeled platform as “lab-intensive” and highlighting the burden of multi-step functionalization protocols [41]. These difficulties were reflected in a wide range of radiochemical yields, ranging from as low as 27% for the bimodal MoonTag-based tracer due to the slow reaction kinetics of its NODAGA chelator [42] to over 85% for CD38-targeting bimodal tracer by using the fast-binding chelator TOHP [53]. In contrast, radiochemical purity after purification was consistently high, often exceeding 95% [44, 49, 52, 53]. Purification was typically achieved using standard chromatographic methods such as size-exclusion chromatography (SEC) or affinity chromatography. However, one study cited difficulties in purifying an IRDye800CW-labeled product from the unreacted nanobody due to their similar molecular weights [41]. 

Beyond synthesis and purification, another critical consideration in bimodal tracer development is the preservation of bioactivity and in vivo stability following functionalization. A unique challenge in bimodal design is the potential for interference between the two imaging labels, which can compromise performance. This can lead to steric hindrance, an effect where the bulky nature of the first label restricts access to nearby conjugation sites, making the attachment of the second label more difficult. For instance, one study reported “radiobleaching” of IRDye800CW, a cyanine-based dye, while being exposed to high activities of ^99m^Tc, necessitating a reduction in radioactivity to preserve the fluorescent signal [41]. Furthermore, in vivo stability issues were also taken into consideration, with one radiolabeled agent showing dissociation of ^99m^Tc, leading to off-target bone accumulation [48] and another study showing potential hydrolysis of the [^64^Cu]Cu-NOTA conjugate over a 24-h period [46]. These findings highlight the need for careful optimization of metal-chelator and fluorophore combinations to ensure stable dual functionalization and tracer integrity over time. Despite these challenges, several publications noted that their functionalized nanobodies retained high binding affinity for their respective targets [40, 44–46, 48, 49, 52].

### Preclinical evaluation and imaging outcomes

Preclinical studies consistently demonstrated effective and specific tumor targeting using nanobody-based tracers, although the extent and kinetics of tracer uptake varied depending on the molecular construct and target (Table 6). Across the reviewed literature, these tracers were developed against a variety of clinically relevant molecular targets, the most common being members of the epidermal growth factor receptor family (EGFR [43, 49, 54] and HER2 [40, 44]), as well as the carcinoembryonic antigen (CEA) [48, 52] and PD-L1 [45, 50] (Fig. 2F). These nanobody tracers were evaluated in various preclinical cancer models, with breast cancer [44, 46, 49, 51], head and neck cancer [43, 47, 49, 54], colorectal cancer [41, 51, 52], and melanoma [45, 46, 50] as the most frequently studied types (Fig. 2E).

**Table 6:** Summary of preclinical performance and imaging outcomes of nanobody-based tracers. Data are presented as mean ± standard deviation where available

Lead compound	Tumor model	Peak tumor uptake [%ID/g]	Time of peak uptake [h p.i.]	Tumor-to-muscle ratio	Ref
2Rs15d	SKOV3 & MDA-MB-435S xenografts	2.2 ± 0.5^**a**^	1	19.8 ± 3.8^**b**^	[40]
uPAR15-GEM	HT-29 xenografts	0.51 ± 0.07^**a**^	4	~ 3.5^**c,d**^	[41]
MoonTag Nb	A4573 xenografts (NSG mice)	1.96 ± 0.83^**e**^	1.5	12.4 ± 2.8^**f**^	[42]
α-EGFR-dPG	A431 xenografts	~ 0.4^**g**^	24	-	[43]
sdAb 7C12- Si-NPs	FaDu cells (in vitro)	n.a	n.a	n.a	[54]
2Rs15d	BT474M1 xenografts	12.00 ± 4.58 (^111^In); 14.07 ± 2.92 (^68^Ga)	1.5	30.70 ± 21.01 (^111^In); 55.58 ± 8.63 (^68^Ga)	[44]
HNI01	LS174T xenografts	9.55 ± 2.86	0.5	-	[52]
KN046	B16F10 syngeneic model	10.21 ± 0.89	72	11.34 ± 0.62^**h**^	[45]
NJB2	Multiple models (TNBC, PDAC and MMTV-PyMT)	-	2.5	6.5-fold > [^18^F]FDG^**i**^	[46]
1E7-Fc	Detroit 562 xenografts	8.4	72	-	[47]
CD3813	H929 and MM.1S xenografts	6.50 ± 2.69 (H929); 2.08 ± 0.28 (MM.1S)	4 (H929); 1 (MM.1S)	96.16 ± 60.62^**j**^	[53]
15.2 m	H460 xenografts	~ 10^**c**^	1	> 10	[48]
D10	A431 and MDA-MB-468 xenografts	2.27 ± 0.68 (A431); 1.30 ± 0.27 (MDA-MB-468)	0.75	36.2 ± 20.9 (A431); 42.8 ± 27.0 (MDA-MB-468)	[49]
K2	624-MEL melanoma xenografts	-	24	-	[50]
NJT6	LM2 TNBC orthotopic and lung metastasis models	4.5 (mammary tumor); 3.5 (lung metastases)	2	-	[51]

^a^Value reported in %ID/cm^3^. ^b^The corresponding fluorescence T/M ratio was 4.6 ± 1.5. ^c^Value estimated from a figure in the source publication. ^d^Value is a tumor-to-background ratio; the corresponding fluorescence ratio was 2.4 ± 0.2. ^e^Ex vivo data. The corresponding in vivo uptake was 1.7 ± 0.5 %ID/mL. ^f^Ex vivo data. The corresponding in vivo T/M ratio was 3.1. ^g^Data reported as standardized uptake value (SUV), not %ID/g. ^h^Value is target-to-normal tissue ratio (TNR). ^i^Value represents the T/M ratio compared to the [^18^F]FDG scan. A ratio for the tracer alone was not provided. ^j^Data for H929 xenografts. A T/M ratio for the MM.1S model was not reported

**Fig. 5:**
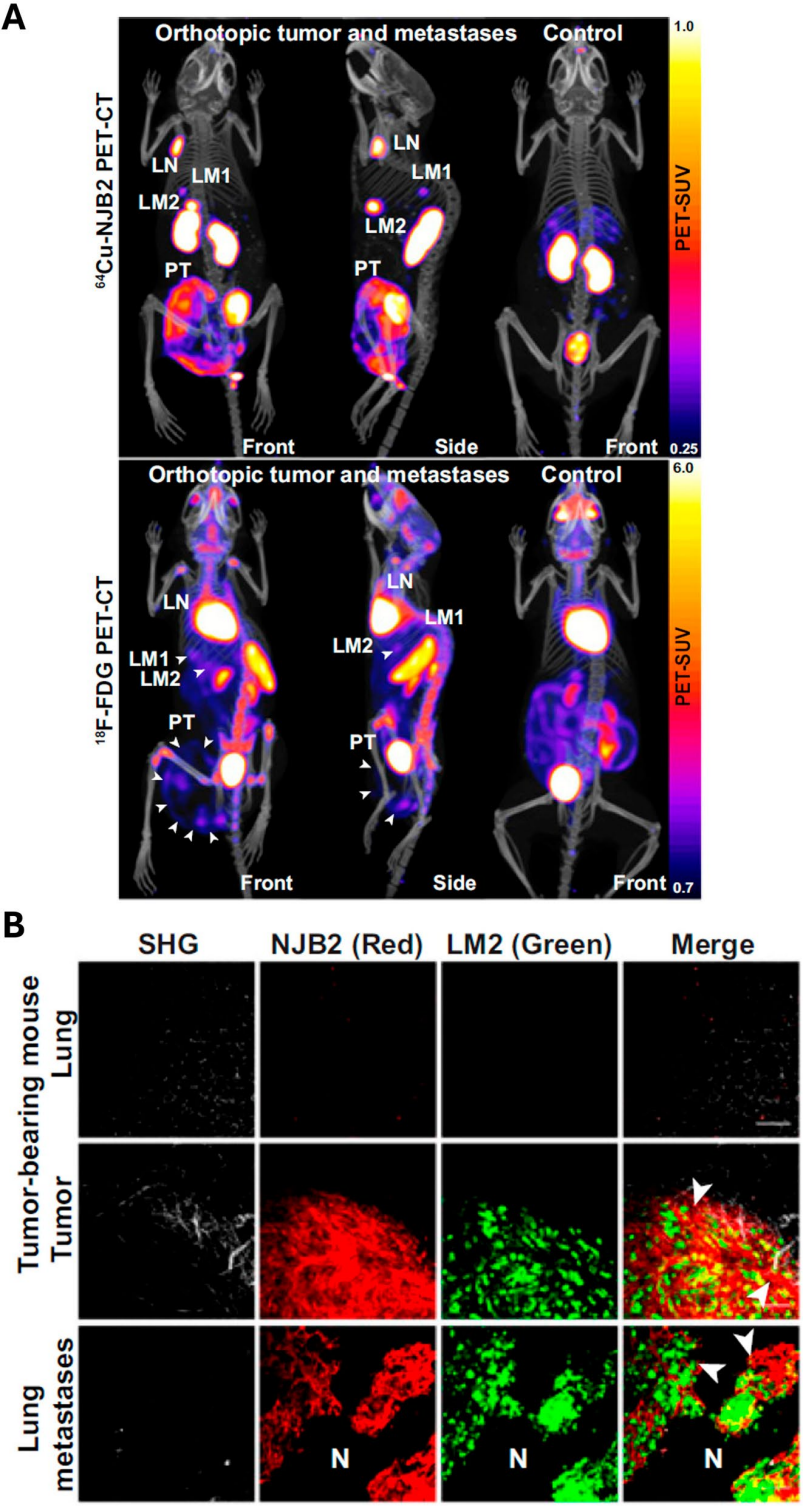
Suitability of the direct labeling strategy for nuclear and optical imaging using the nanobody NJB2. **A)** Representative PET/CT imaging comparison of the same NSG mice bearing metastatic breast cancer tumors imaged with [^64^Cu]Cu-NJB2 or [^18^F]FDG. [^64^Cu]Cu-NJB2 achieves superior signal-to-noise ratios (top) compared to the metabolic tracer [^18^F]FDG (bottom), allowing for the clear visualization of discrete lymph node (LN) and liver metastases (LM1, LM2) that are poorly defined by [^18^F]FDG. **B)** Validation of targeting specificity using the optical equivalent of the tracer (NJB2-Texas Red). Imaging with two-photon microscopy two hours after injection demonstrates intense accumulation of NJB2-Texas Red (red) in the extracellular matrix of primary tumors (middle row) and lung metastases (bottom row), where it colocalizes with ZsGreen-expressing tumor cells (green). Importantly, no uptake is observed in the normal lung tissue (top row) and extracellular matrix of normal tissue (marked as “N”), confirming the high specificity of the direct labeling strategy. (Scale bars, 100 μm) These images were adapted from Jailkhani N, Ingram JR, Rashidian M, Rickelt S, Tian C, Mak H, et al. Noninvasive imaging of tumor progression, metastasis, and fibrosis using a nanobody targeting the extracellular matrix. Proceedings of the National Academy of Sciences. 2019;116(28):14,181–90

Several tracers were particularly effective, achieving high tumor accumulation at early time points. For example, the HER2-targeting [^68^Ga]Ga-2Rs15d reached an uptake of 14.07 ± 2.92%ID/g at just 1.5 h post-injection in a subcutaneous BT474M1 breast cancer xenograft model [44], while the CEA-targeting [^68^Ga]Ga-HNI01 peaked at 9.55 ± 2.86%ID/g in just 30 min in subcutaneous LS174T colorectal cancer xenografts [52]. In contrast, a number of tracers were designed for long circulation time and delayed imaging, often involving long-lived nuclides and larger constructs. A key example is the bispecific nanobody-tracer [^131^I]I-KN046, which incorporates nanobody domains targeting both PD-L1 and cytotoxic T-lymphocyte-associated protein 4 (CTLA-4) [45]. Due to its large size, it reached its maximum tumor uptake of 10.21 ± 0.89%ID/g at 72 h post-injection in a subcutaneous B16F10 syngeneic melanoma model [45]. Similarly, the MET (receptor tyrosine kinase MET)-targeting tracer [^89^Zr]Zr-1E7-Fc, a nanobody fused to an antibody Fc-domain to deliberately extend its half-life, also followed this slow kinetic profile, showing a high tumor uptake of 8.4%ID/g at the 72-h time point in subcutaneous head and neck cancer xenografts derived from Detroit 562 cells [47]. The specificity of this targeting was a key validation point in many of the reviewed studies, typically demonstrated using blocking experiments or non-targeting control nanobodies. This high level of specific uptake translated directly into high-contrast imaging, as evidenced by the excellent tumor-to-muscle ratios reported for many of the tracers (Table 6). Tumor-to-muscle ratio is a key factor determining clinical utility, with a value > 5 often considered suitable for diagnostic imaging and a value > 10 being desirable for image-guided therapy [56, 57]. The cluster of differentiation 38 (CD38) targeting nanobody [^68^Ga]Ga-TOHP-CD3813 produced images with remarkable contrast, achieving a high mean tumor-to-muscle ratio of 96.16 at 4 h post-injection in subcutaneous H929 xenografts, though the results showed significant variability between subjects (SD = 60.62) [53]. The HER2-targeting [^68^Ga]Ga-2Rs15d resulted in a tumor-to-muscle ratio of 55.58 ± 8.63 at just 1.5 h post-injection in a BT474M1 breast cancer xenograft model [44]. Other radiolabeled agents, such as the EGFR-targeting [^99m^Tc]Tc-D10 and the CEA-targeting [^99m^Tc]Tc-15.2m showed tumor-to-muscle ratios exceeding 36 in subcutaneous A431 xenografts at 45 min [49], and 10 within 1 h in subcutaneous H460 xenografts [48], respectively. A key finding from one study was the outperformance of the extra domain-B of fibronectin (FN-EIIIB)-targeting radiolabeled agent [^64^Cu]Cu-NJB2 compared to the clinical standard [^18^F]FDG (Fig. 5A) [46]. It achieved a tumor-to-muscle ratio 6.5-fold higher than that of the standard in a model of lymph node metastasis, demonstrating the potential for this specific approach to provide notably high-contrast images. This high-contrast visualization was a consistent finding across the reviewed studies, as further evidenced by the specific tumor uptake observed in both direct (Fig. 3B) [40] and scaffold-mediated (Fig. 4B) [42] PET/SPECT imaging results.

In terms of biodistribution, a consistent finding across preclinical studies for the conventional, small-sized nanobody tracers was the rapid, predominant renal clearance. While this fast clearance contributed to the high tumor-to-background contrast, it also resulted in extremely high tracer accumulation and retention in the kidneys. For example, the EGFR-targeting radiolabeled agent [^99m^Tc]Tc-D10 showed a kidney uptake of 160.7 ± 17.9%ID/g, a value approximately 70 times higher than its corresponding tumor uptake at the same 45-min post-injection time point [49]. Similarly, the uPAR-targeting tracer uPAR15-GEM-[^99m^Tc]Tc(CO)_3_-IRDye800CW showed rapid renal elimination reaching 188 ± 24%ID/g just one hour after injection [41]. This prominent renal signal was frequently noted as a potential limitation that could obscure the imaging of nearby lesions and was highlighted as a key consideration to improve the clinical translation potential due to the undesired associated radiation dose to the kidneys.

The fluorescence imaging performance of the developed probes was also evaluated across the studies, with three studies reporting quantitative contrast metrics. While the anti-HER2 “multifunctional single attachment point" (MSAP) tracer achieved a fluorescent tumor-to-muscle ratio of 4.6 [40] and the anti-uPAR GEM tracer showed a tumor-to-background ratio of 2.4 [41], a dPG scaffold-based tracer demonstrated nearly fivefold higher fluorescence within the tumor compared to its non-targeting control [43]. Qualitatively, multiple studies described high-contrast and specific tumor visualization [40, 41, 46, 50, 51], as depicted by the representative fluorescence imaging results (Fig. 3B) [40] and microscopy validation (Fig. 5B) [46] of the nanobody-based systems. This optical performance translated directly to surgical relevance by enabling the accurate fluorescence-guided removal of submillimeter tumor lesions in one of the studies [40]. This study also demonstrated a strong correlation for the bimodal construct, with a correlated R^2^ value of 0.97 between the SPECT and fluorescence signals in resected tumors [40]. Finally, the choice of fluorophore was highlighted as a critical design parameter. While several studies noted the advantages of NIR dyes for tissue penetration compared to conventional visible-spectrum probes [40, 41, 43, 46], one study reported a "drastic detrimental effect" on pharmacokinetics from the random conjugation of the hydrophobic dye IRDye800CW [41].

### Translation to clinical settings

Of the 15 tracers included in this review, two have progressed from preclinical models to early-phase, first-in-human clinical trials: the CEA-targeting tracer [^68^Ga]Ga-HNI01 [52] and the CD38-targeting tracer [^68^Ga]Ga-TOHP-CD3813 [53]. These studies provide the first insights into the safety, biodistribution, and imaging performance of these nanobody-based tracers in patients. It is important to note that both trials evaluated the mono-modal, radiolabeled forms of the nanobodies. While they do not assess bimodal performance, they provide essential data on safety and renal dosimetry of these specific nanobody scaffolds, establishing the foundation for the future translation of dual-labeled variants.

**Fig. 6:**
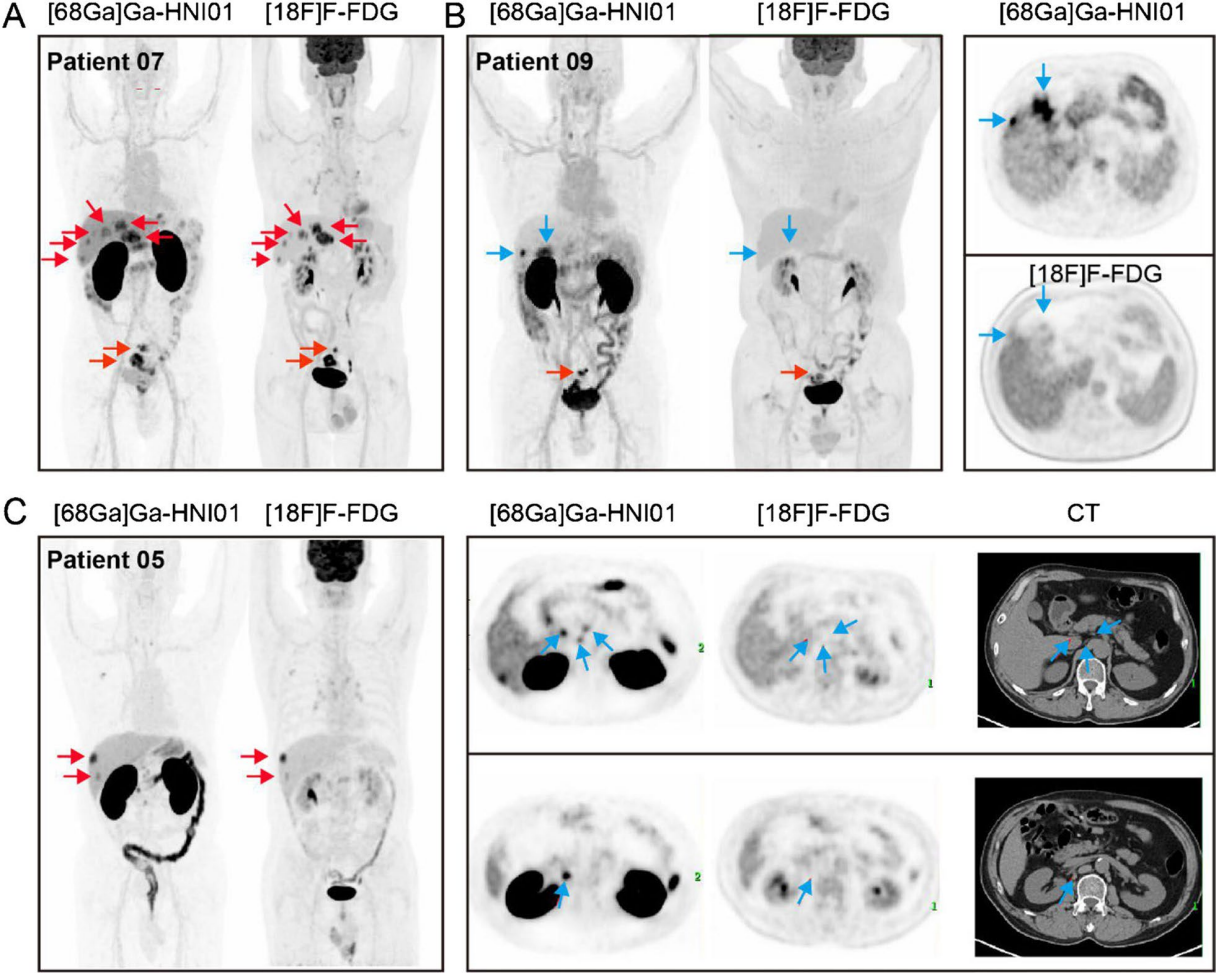
Comparative PET imaging of a CEA-targeting nanobody, [^68^Ga]Ga-HNI01, and [^18^F]F-FDG in colorectal cancer metastases.** A)** MIP images from [^68^Ga]Ga-HNI01 PET/CT (left, at 2 h p.i.) and [^18^F]FDG PET/CT (right) in a patient with liver metastases and primary lesions. [^68^Ga]Ga-HNI01 PET/CT and [^18^F]FDG PET/CT both detected the primary and hepatic lesions (red arrow), indicating the ability of [^68^Ga]Ga-HNI01 to detect liver metastasis of colorectal cancer (CRC). **B)** The MIP (left) and transaxial (right) PET images in a second patient showed that the liver metastases (blue arrow) exhibited only high uptakes of [^68^Ga]Ga-HNI01, whereas [^18^F]FDG was negative. The primary lesions (red arrow) of patient 09 were detected by both tracers. **C)** The MIP (left) and transaxial (right) PET images in a third patient. The MIP images showed that hepatic lesions (red arrow) were clearly detected by both tracers. However, the lymph node metastases (blue arrow) in the liver were seen only by [^68^Ga]Ga-HNI01 and showed low [^18^F]FDG accumulation. This image was adapted from Li L, Lin X, Wang L, et al. Immuno-PET of colorectal cancer with a CEA-targeted [^68^Ga]Ga-nanobody: from bench to bedside. Eur J Nucl Med Mol Imaging. 2023;50(13):3735–49

The CEA-targeting tracer, [^68^Ga]Ga-HNI01, was evaluated in nine patients with colorectal cancer [52]. This tracer was reported to be well-tolerated with no adverse events, and dosimetry analysis revealed that the kidneys received the highest absorbed dose (0.2770 ± 0.0584 mGy/MBq), which is below kidney dose reported for other established ^68^Ga-based agents like [^68^Ga]Ga-PSMA-11 (0.24 ± 0.04 mGy/MBq) [58]. The agent demonstrated excellent imaging capabilities, visualizing lesions with high contrast as early as 30 min post-injection and achieving a mean maximum standardized uptake value (SUVmax) of 11.49 in primary tumors at 2 h post-injection. In a direct comparison with [^18^F]FDG PET/CT, [^68^Ga]Ga-HNI01 successfully identified six liver metastases that were not detected by the clinical standard (Fig. 6). However, a key challenge reported was the strong, abnormal uptake in the healthy colon and rectum of five patients.

The second agent, the CD38-targeting [^68^Ga]Ga-TOHP-CD3813, was studied in two patients with multiple myeloma and was also found to be safe, with no significant adverse effects [53]. This study reported clear visualization of multiple myeloma lesions with a mean SUVmax of 5.99, and its dosimetry estimates indicated that the kidneys received the highest dose (0.367 mSv/MBq). Notably, it was also reported that [^68^Ga]Ga-TOHP-CD3813 detected more lesions with higher contrast than [^18^F]FDG.

## Discussion and outlook

Following a systematic literature search that identified 514 records and resulted in the screening of 293 unique articles, the final analysis of the 15 peer-reviewed articles that met the inclusion criteria provides an overview of the progression of nanobody-based tracers potentially amenable for dual-modality imaging, from foundational concepts to the first stages of clinical evaluation. The findings demonstrate that while this approach shows great promise in preclinical models, often achieving high-contrast tumor imaging superior to the clinical standard, its development and eventual clinical impact is still hampered by significant hurdles related to chemical synthesis, functionalization and high renal uptake, as also reflected in the first clinical data obtained with the CEA-targeting tracer [^68^Ga]Ga-HNI01 [52] and the CD38-targeting tracer [^68^Ga]Ga-TOHP-CD3813 [53].

Despite the relatively early stage of the bimodal nanobody tracer field, this analysis already allows us to identify some trends in several aspects ranging from the design to their initial clinical testing. Specifically, this discussion will first analyze the reported design strategies and the comparative advantages of the nanobody platform. It will then critically evaluate the key chemical and biological hurdles identified in this review, before addressing the limitations in study comparability and concluding with a future outlook for the field.

### a. Design of bimodal nanobody-based tracers

The finding that a majority of the included studies (10 out of 15) used independently labeled tracers for comparative purposes, suggests that for many, the complexity and “lab-intensive” nature of engineering simultaneous bimodal constructs remains a significant technical barrier, as evidenced by the chemical challenges mentioned in some reviewed studies [40, 41, 44, 49, 51, 53, 54]. While stepwise validation of individual components is a more practical and reliable approach, it also sacrifices the benefits of a true bimodal tracer. The key benefit of a dual-labeled tracer is the certainty of signal co-localization, ensuring a direct correlation between the preoperative scan and the intraoperative visualization [9]. This is an advantage over co-administering two separate tracers, as it eliminates any potential for pharmacokinetic differences or mismatched signals [59]. This certainty of co-localization is most valuable in complex procedures like debulking surgery, where it gives the surgeon high confidence that the visual fluorescent signal corresponds to a PET-positive lesion, justifying the resection of small tumors that are difficult to identify by sight or palpation alone [60].

However, despite the limitation regarding simultaneous signal co-localization, the studies utilizing independent labeling strategies hold significant value as necessary preliminary steps in the development of bimodal tracers. These investigations serve as crucial "proof-of-concept" verifications, demonstrating the fundamental compatibility between the targeting nanobody and the distinct imaging modalities (radionuclides and fluorophores). By demonstrating that the nanobody scaffold retains its stability and binding affinity when conjugated to either modality individually, these studies effectively validate the essential "components" required for a bimodal system. Consequently, future research should prioritize merging these validated components into single bimodal constructs, utilizing the efficient modular conjugation strategies discussed in this review.

To address the challenge of attaching multiple labels to the small nanobody molecule, some researchers have utilized more complex scaffold-based platforms, including dendritic polymers or nanoparticles [42, 43, 54]. However, while these larger scaffolds offer a solution for increasing the tracer’s payload capacity, they can also introduce additional manufacturing complexities and regulatory hurdles (e.g., scale-up limitations) and negatively affect the pharmacokinetics of the nanobody, limiting their targeting efficacy [61, 62]. This highlights the need for alternative strategies that can offer bimodality without significantly increasing the size of the final construct. For example, the development of small, pre-functionalized linkers that carry both imaging labels is a promising potential solution, with the MSAP linker used in one of the reviewed studies serving as a prime example of this approach [40]. This strategy could potentially standardize the labeling of a wide range of nanobodies into a single, more efficient step. Crucially, while the pharmacokinetic impact of any modification must still be carefully evaluated, such a standardized method is essential for achieving the scalability and batch-to-batch reproducibility required for clinical manufacturing and regulatory approval [63].

The impact of these different design strategies is directly reflected in the fluorescence imaging performance of the agents. Although quantitative fluorescence data was less frequently reported than for the radiolabeled components, the available metrics were significant for assessing the clinical potential. For example, a tumor-to-muscle ratio of 4.6 reported in one of the studies proved sufficient to enable the fluorescence-guided resection of submillimeter tumor lesions, a key benchmark for clinical utility [40]. This performance directly addresses the historical concern regarding the sensitivity mismatch between nuclear (picomolar) and optical (micro- to nanomolar) imaging [60]. Crucially, studies developing true bimodal constructs provided evidence that this limitation can be addressed by modern tracer design, with one study reporting a near-perfect linear correlation (R^2^ = 0.97) between the SPECT and fluorescence signals from a single injection [40]. This feasibility mirrors established findings in the field of bimodal antibodies, where tracers such as ^111^In-DOTA-girentuximab-IRDye800CW have successfully demonstrated that simultaneous nuclear and optical imaging is achievable in clinical settings [64], further reinforcing the core principle of using a single bimodal tracer to link preoperative scans to real-time intraoperative visualization. Furthermore, the choice of fluorophore and its conjugation strategy emerged as a critical design parameter with an impact on tracer performance. The same study also reported a "drastic detrimental effect" on pharmacokinetics from the random conjugation of the hydrophobic dye IRDye800CW [41], while a site-specific approach with the more hydrophilic Cy5 was highly successful [40]. This underscores that the physicochemical properties of the fluorescent probe and its attachment method can significantly influence the tracer’s overall biodistribution and require careful optimization.

### b. Key synthetic and biological challenges

The development of nanobody-based tracers is often hampered by substantial technical challenges related to synthesis, labeling control, and tracer stability, with the dual functionalization of nanobodies often being a "lab-intensive" process [40, 41, 44, 48, 52]. Purification of the final tracer represents another key, often underreported, challenge. A clear choice exists between simpler purification methods like SEC, which may yield impure products, and more complex, multi-step strategies that achieve high purity at the expense of yield and scalability [65, 66].

Beyond the chemical synthesis, this review also highlights that in vivo performance faces two distinct biological hurdles. The first is related to the target itself. High tracer specificity, for example, does not always guarantee clinical applicability if the biological target is not exclusive to cancer, as was reported for the FN-EIIIB tracer [46], which could not distinguish cancer from fibrosis. The second, and most critical, translational challenge was high and persistent renal accumulation [40–43, 46–49, 52, 53]. The fact that high renal uptake was consistently reported in preclinical models and confirmed as the primary dose-limiting factor in first-in-human trials underscores the need to address kidney retention before nanobody-based tracers can achieve widespread clinical use as systemic agents.

### c. Limitations regarding comparability across reported studies

While this systematic review provides valuable insights into nanobody-based bimodal imaging tracers, certain considerations should be acknowledged. The included studies exhibited a high degree of heterogeneity in terms of tracer design, molecular targets, radionuclides, and preclinical models. Specifically, the variability in bioconjugation strategies acts as a major source of heterogeneity, as the chemical modification method heavily influences nanobody affinity and pharmacokinetics. Additionally, while the radionuclide uptake can be standardized, fluorescence intensity is highly dependent on instrumentation settings, tissue depth, and the photophysical properties of the dye, making the direct cross-study comparison of optical performance difficult. This issue is further complicated by the predominant use of visible spectrum fluorophores in a significant number of studies. While these dyes are effective for confirming nanobody targeting specificity in preclinical models, their poor tissue penetration makes them unsuitable for clinical intraoperative navigation, limiting the translational impact of these specific studies to proof of targeting feasibility rather than validations of a clinically applicable imaging protocol.

Consequently, a quantitative meta-analysis was not feasible, necessitating a narrative synthesis of the results to best capture the breadth of the available evidence. Furthermore, as with any literature-based review, the possibility of publication bias cannot be fully excluded, as studies reporting negative or null results may be underrepresented. Finally, the risk of bias assessment revealed a predominance of “unclear” risks across the included studies. This finding highlights a general lack of standardized reporting in preclinical research within this field, rather than inherent methodological flaws, underscoring the need for more rigorous reporting guidelines to better guide the future clinical translation.

Finally, a significant gap remains in the translational evidence: the clinical trials described in this review evaluated only mono-modal radiolabeled nanobodies. Consequently, no clinical data currently exists regarding the safety or intraoperative efficacy of the fluorescent component for these tracers, and conclusions regarding their surgical utility remain derived from preclinical findings and the comparative clinical data derived from bimodal antibodies and peptides.

### d. A comparative analysis of bimodal targeting molecules

To contextualize the findings of this review, it is important to compare the performance of the nanobody platform against other targeting strategies based on antibodies and peptides, as summarized in Table 2. The findings of this review highlight how the size of nanobodies (~ 15 kDa) dictates a distinct and highly tunable pharmacokinetic profile. Although this profile can be deliberately slowed through protein engineering, the conventional nanobody platform is defined by its rapid kinetics [24]. While bimodal tracers based on full-sized monoclonal antibodies (~ 150 kDa) have been developed, their slow kinetics and long circulation half-life require multi-day imaging protocols (typically 3–7 days) for sufficient background clearance [20, 67]. This long imaging window presents a significant challenge for the rapid, same-day workflow required in surgical guidance, and typically results in higher background signal.

At the other end of the size spectrum, small peptide-based bimodal tracers (< 5 kDa) offer the advantage of very rapid clearance, which is beneficial for minimizing background signal [21, 68]. However, their very fast washout can prevent sufficient tumor retention needed for intraoperative applications, with reported uptakes often in the 1–5%ID/g range [21, 69]. The relatively high tumor uptakes reported for nanobodies (frequently exceeding 9%ID/g) [44, 45, 48, 52] suggest that they may offer a more favorable balance between fast systemic clearance and effective tumor retention for high-contrast imaging.

Overall, the unique balance of the pharmacokinetic properties of nanobodies between antibodies and peptides positions them as a promising tool for applications requiring high-contrast images on the same day of administration. This is particularly valuable for intraoperative surgical guidance, where rapid tumor penetration and rapid background clearance are crucial.

### e. Clinical translation and future outlook

Bimodal tracers have the potential to address the existing limitations in image-guided surgical oncology, which, to date, cannot be fully met by current single-modality tracers. Currently, surgeons rely on a range of established radiolabeled agents and optical probes to guide resections (Table 7) [30, 70]. Fluorescence imaging takes advantage of greater NIR light tissue penetration and a higher signal-to-background ratio and utilizes general, non-specific perfusion dyes like indocyanine green (ICG) [71] as well as targeted molecules such as Pafolacianine [78], a folate-conjugated dye which binds to folate receptors on cancer cells, or the novel investigational dye IRDye800CW [29]. In contrast, the frequent use of the visible-spectrum dye FITC in several of the reviewed studies [45, 48, 52–54] likely reflects its role as a convenient and cost-effective label for early-stage, proof-of-concept experiments.

**Table 7:** Clinically established single-modality tracers for surgical guidance

Fluorescent probes	Emission wavelength [nm]	Mechanism/ Target	Primary surgical application	Ref	
Indocyanine green (ICG)	~ 830	Perfusion / Enhanced permeability and retention (EPR) effect	Perfusion imaging; Sentinel lymph node detection	[71, 72]	
Methylene blue	~ 686	Non-specific tissue staining	Sentinel lymph node mapping; Ureter visualization	[72, 73]	
5-Aminolevulinic acid (5-ALA)	~ 635	Metabolic conversion to Protoporphyrin IX in tumor cells	Intraoperative imaging of malignant gliomas	[74, 75]	
Fluorescein sodium	~ 538	Perfusion / Enhanced permeability and retention (EPR) effect	Neurosurgery (e.g., glioma resection)	[76, 77]	
Pafolacianine (Cytalux®)	~ 796	Folate receptor	Ovarian and lung cancer surgery	[78, 79]	
Pegulicianine (Lumisight™)	~ 675	Cathepsin proteases	Breast cancer surgery (lumpectomy)	[80, 81]	

Radiolabeled agents	Radionuclide	Imaging modality	Mechanism/ Target	Primary surgical application	Ref
[^99m^Tc]-Nanocolloid	^99m^Tc	SPECT	Lymphatic flow (non-specific)	Sentinel lymph node detection	[82]
[^99m^Tc]-Tilmanocept (Lymphoseek®)	^99m^Tc	SPECT	CD206 on macrophages	Sentinel lymph node detection	[83]
[^99m^Tc]-Sestamibi (MIBI)	^99m^Tc	SPECT	Mitochondrial membrane potential	Intraoperative detection of parathyroid adenomas	[84]
[^111^In]-Pentetreotide (Ostreoscan®)	^111^In	SPECT	Somatostatin receptors (SSTR2)	Neuroendocrine tumors (off-label surgical guidance)	[85]

A parallel development has occurred in nuclear-guided surgery, where non-specific agents like (^99m^Tc)-Nanocolloid [82] for mapping lymph flow are now complemented by highly specific agents like (^111^In)-Pentetreotide [85] that target somatostatin receptors on neuroendocrine tumors. A prime example of this targeted approach is the use of PSMA-targeting radiolabeled agents, such as widely used [^68^Ga]Ga-PSMA-11, in high-risk prostate cancer to detect metastatic lymph nodes, which is essential for guiding the extent of the subsequent surgical resection [86]. The critical limitation common to these agents, however, is their reliance on separate tracers for separate imaging modalities, which are unable to provide complementary nuclear and optical information simultaneously.

The preclinical and clinical outcomes reported in this review demonstrate the clear potential of bimodal nanobody tracers to bridge this gap. The consistent selection of typically overexpressed oncological targets such as HER2 [40, 44], EGFR [43, 49, 54], and CEA [48, 52], shows a strong focus on clinical relevance. The high tumor-to-muscle ratios achieved by several tracers, with values exceeding 96 for a CD38-targeted agent [53] and 55 for a HER2-agent [44], provide strong evidence for the platform’s potential in applications requiring high sensitivity, such as intraoperative tumor margin delineation. This is further underscored by the finding that several tracers directly outperformed the clinical standard, [^18^F]FDG, either by detecting lesions that [^18^F]FDG missed or by achieving significantly higher tumor-to-background contrast [46, 52, 53].

Looking ahead, translating the promising preclinical findings into routine clinical practice will require focused progress in several key areas. First, advances in the chemical engineering of nanobody-based tracers themselves are needed to overcome the current “lab-intensive” and complex functionalization strategies by developing more efficient, scalable, and modular labeling systems. The second, and perhaps more critical, area is the biological optimization of the in vivo performance of the tracer. Successfully mitigating the high renal uptake that was consistently observed in both preclinical and clinical studies is essential for the future success of nanobody-based platforms as a systemically administered agent. This will require dedicated research into strategies such as the co-infusion of plasma expanders like Gelofusine [24, 87] or the further development of engineered nanobodies with modified charges or cleavable linkers designed to release the radionuclide from the kidney [24]. Finally, once these chemical and biological hurdles are addressed, well-designed early-phase clinical trials will be essential to evaluate safety, pharmacokinetics, and diagnostic performance, ultimately evaluating the clinical impact of these agents for cancer imaging and intraoperative guidance.

Beyond diagnostics, the modularity of the nanobody platform also makes it highly suitable for theranostic applications. By swapping a diagnostic radionuclide like ^68^Ga or ^89^Zr with a therapeutic one like lutetium-177 (^177^Lu) or actinium-225 (^225^Ac), the same bimodal tracer could be used first for PET imaging and surgical guidance, and then for targeted radionuclide therapy to destroy residual cancer cells. A similar modular approach applies to fluorescent dyes, where the switch from diagnostically preferable labels, such as ICG, to therapeutically active NIR dyes, such as IR700 phthalocyanine, open opportunities for photodynamic therapy [88]. Together, these features highlight the potential of these tracers to combine diagnosis and therapy in a single platform, towards developing nanobody-based bimodal theranostic agent.

## Conclusion

This systematic review highlights bimodal nanobody-based tracers as a promising platform for cancer imaging with particular potential for surgical guidance. The preclinical findings across the 15 included studies showcase the value of these tracers, with the first results of dual-functionalized nanobodies demonstrating strong tumor targeting and high imaging contrast, and several tracers also showing advantages over the current clinical standard, [^18^F]FDG. These findings underscore the potential of a single, bimodal tracer to enhance surgical precision and improve oncologic outcomes by simultaneously providing deep-tissue localization and real-time visual feedback. However, their successful clinical translation will depend on overcoming two key challenges. First, standardized and efficient methods for tracer synthesis are needed to ensure reproducibility and stability, and to guarantee that the functionalization process does not compromise the nanobody’s targeting efficiency or favorable pharmacokinetic profile. Second, and more critically, strategies must be developed to optimize biodistribution by reducing renal accumulation, a limitation observed across multiple studies. Targeted efforts to resolve these chemical and biological hurdles will be essential to unlock the clinical utility of this promising imaging platform, and they could enable the future development of nanobody platforms as theranostic agents for combined imaging and therapy.

## Supplementary Information

Additional file 1.

## Data Availability

Data sharing is not applicable to this article as no datasets were generated or analyzed during the current study.

## Abbreviations

5-ALA: 5-Aminolevulinic acid
CD: Cluster of differentiation
CEA: Carcinoembryonic antigen
CT: Computed tomography
CTLA-4: Cytotoxic T-lymphocyte-associated protein 4
Cy3 (5/7): Cyanine dye 3 (5/7)
dPG: Dendritic polyglycerol
DFO: Deferoxamine
DMPTACN: 1,4-Bis-(2-pyridinylmethyl)-1,4,7-triazacyclononane
DOTA: 1,4,7,10-Tetraazacyclododecane-1,4,7,10-tetraacetic acid
DTPA: Diethylenetriaminepentaacetic acid
EDB: Extra domain B
EGFR: Epidermal growth factor receptor
EPR: Enhanced permeability and retention
FDG: 2-Fluoro-2-deoxy-D-glucose
FITC: Fluorescein isothiocyanate
FN-EIIIB: Type III extra domain-B of fibronectin
HER2: Human epidermal growth factor receptor 2
ICG: Indocyanine green
IRDye800CW: Infrared dye 800CW
MET: Receptor tyrosine kinase MET
MSAP: Multifunctional single attachment point
MW: Molecular weight
NIR: Near-infrared
NODAGA: 1,4,7-Triazacyclononane-1-glutaric acid-4,7-diacetic acid
NOTA: 1,4,7-Triazacyclononane-1,4,7-triacetic acid
NP: Nanoparticle
PD-L1: Programmed death-ligand 1
PET: Positron emission tomography
RES: Reticuloendothelial system
SD: Standard deviation
SEC: Size-exclusion chromatography
SPECT: Single-photon emission computed tomography
SSTR2: Somatostatin receptors
SUV: Standardized uptake value
THP: Tris(hydroxypyridinone)
TNC: Tenascin C
uPAR: Urokinase plasminogen activator receptor
