# Supplementary material for: Bimodal nanobody agents for cancer imaging and potential intraoperative guidance: a systematic review
Source: J Nanobiotechnology. 2026 Mar 3;24:329. doi: 10.1186/s12951-026-04192-w (PMC13064313; doi:10.1186/s12951-026-04192-w)
Supplement: Supplementary file 1 — Additional file 1. [file 12951_2026_4192_MOESM1_ESM.docx]

**Risk of Bias Analysis (SYRCLE tool)**

The methodological quality of the 12 preclinical studies was assessed using the SYRCLE tool, an adaptation of the Cochrane RoB tool for animal studies. This tool covers ten domains:

- Sequence Generation (Random allocation)
- Baseline characteristics
- Allocation concealment
- Random housing
- Blinding of researchers
- Random outcome assessment
- Blinded outcome assessment
- Incomplete outcome data
- Selective outcome reporting
- Other sources of bias

Each domain is rated as:

- low risk
- unclear risk
- high risk

**Debie et al. (2023)**

Title: The design and preclinical evaluation of a single-label bimodal nanobody tracer for image-guided surgery.

- **Animal Model:** Xenograft models of ovarian cancer (SKOV3 and MDA-MB-435S) in nude mice.
- **Assessment:** Randomization, allocation concealment and blinding details are not explicitly stated → selection and detection bias domains "Unclear". The risk for incomplete outcome data and selective outcome reporting is considered "Low", as all animals appear to be accounted for in the results.

**Declerck et al. (2023)**

Title: The GEM-handle as convenient labeling strategy for bimodal single-domain antibody-based tracers carrying ^99m^Tc and a near-infrared fluorescent dye for intraoperative decision-making.

- **Animal Model:** HT-29 colorectal cancer xenograft model in nude mice.
- **Assessment:** Key details on randomization and allocation concealment were provided → "Low" risk for selection bias. Blinding was not specified → detection and performance bias "Unclear". Data completeness was high → incomplete and selective reporting rated “Low”.

**Höffgen et al. (2024)**

Title: Combining poly-epitope MoonTags and labeled nanobodies for signal amplification in cell-specific PET imaging *in vivo.*

- **Animal Model:** A4573 tumor xenograft model in NSG mice.
- **Assessment:** Baseline characteristics were well-described → "Low" risk. No mention of randomization, concealment, or blinding → other key domains "Unclear". No suggestion of incomplete data → "Low" risk for that domain.

**Pant et al. (2020)**

Title: Active targeting of dendritic polyglycerols for diagnostic cancer imaging.

- **Animal Model:** A431 epidermoid carcinoma xenograft model.
- **Assessment:** No description of randomization, blinding, or allocation concealment → key domains "Unclear". The risk for incomplete outcome data was also "Unclear", while selective reporting was "Low".

**Massa et al. (2016)**

Title: Sortase A-mediated site-specific labeling of camelid single-domain antibody-fragments: a versatile strategy for multiple molecular imaging modalities.

- **Animal Model:** HER2-expressing BT474M1 breast cancer xenograft model.
- **Assessment:** No explicit randomization or blinding was described → key domains "Unclear". No obvious issues with missing data → incomplete and selective reporting rated "Low".

**Jiang et al. (2023)**

Title: Enhanced antitumor immune responses via a new agent [^131^I]‑labeled dual‑target immunosuppressant.

- **Animal Model:** Syngeneic B16F10 melanoma model.
- **Assessment:** Key details on randomization, allocation concealment and blinding were missing → selection and detection bias domains "Unclear”. The data reporting was complete → "Low" risk for incomplete outcome data and selective reporting.

**Jailkhani et al. (2019)**

Title: Noninvasive imaging of tumor progression, metastasis, and fibrosis using a nanobody targeting the extracellular matrix.

- **Animal Model:** Multiple models, including TNBC xenografts and genetically engineered KPC and MMTV-PyMT mice.
- **Assessment:** No details on randomization, blinding, or allocation concealment → "Unclear" risk for these domains. All animals appear to be accounted for across the different models → "Low" risk for incomplete and selective reporting.

**Minne et al. (2024)**

Title: Evaluation of a novel MET-targeting camelid-derived antibody in head and neck cancer.

- **Animal Model:** Human MET-expressing HNSCC xenograft model (Detroit 562).
- **Assessment:** No information on randomization, allocation concealment or blinding → "Unclear" risk for selection and detection bias. The reporting of animal numbers was consistent → "Low" risk for incomplete outcome data.

**Wang et al. (2017)**

Title: A nanobody targeting carcinoembryonic antigen as a promising molecular probe for non‑small cell lung cancer.

- **Animal Model:** Human large cell lung cancer xenograft model (H460) in nude mice.
- **Assessment:** The information on randomization, allocation concealment, or blinding procedures for the in-vivo experiments was not included → the risk of bias is "Unclear". The study accounted for all animals and outcomes, resulting in a "Low" risk for incomplete and selective reporting.

**Krüwel et al. (2016)**

Title: *In vivo* detection of small tumour lesions by multi-pinhole SPECT applying a ^99m^Tc-labelled nanobody targeting the Epidermal Growth Factor Receptor.

- **Animal Model:** Xenograft models of epidermoid carcinoma (A431) and mammary carcinoma (MDA-MB-468) in nude mice.
- **Assessment:** Baseline characteristics well-described → "Low" risk. No mention of a randomization method, blinding and allocation concealment → these key domains are "Unclear". No suggestion on incomplete data → “Low” risk for that domain.

**Heremans et al. (2024)**

Title: Sustained release of a human PD-L1 single-domain antibody using peptide-based hydrogels.

- **Animal Model:** Human melanoma xenograft model (624-MEL).
- **Assessment:** Randomization, allocation concealment, and blinding details are not explicitly stated → key selection and detection bias domains "Unclear". The risk for incomplete outcome data and selective reporting is "Low", since all animals appear to be accounted for in the results.

**Jailkhani et al. (2023)**

Title: Proteomic profiling of extracellular matrix components from patient metastases identifies consistently elevated proteins for developing nanobodies that target primary tumors and metastases.

- **Animal Model:** Multiple models, including TNBC xenografts in NSG mice and a 4T1 syngeneic TNBC model in BALB/c mice.
- **Assessment:** Paper does not detail randomization, blinding, or allocation concealment → key domains "Unclear". Reporting of animal numbers was consistent → "Low" risk for incomplete and selective reporting.

**Risk of Bias Table (SYRCLE)**

| **SYRCLE domain** | **Debie et al. (2021)** | **Declerck et al. (2023)** | **Höffgen et al. (2024)** | **Pant et al. (2020)** | **Massa et al. (2016)** | **Jiang et al. (2023)** | **Jailkhani et al. (2019)** | **Minne et al. (2024)** | **Wang et al. (2017)** | **Krüwel et al. (2016)** | **Heremans et al. (2024)** | **Jailkhani et al. (2023)** |
| --- | --- | --- | --- | --- | --- | --- | --- | --- | --- | --- | --- | --- |
| Sequence generation | Unclear | Low | Unclear | Unclear | Unclear | Unclear | Unclear | Unclear | Unclear | Unclear | Unclear | Unclear |
| Baseline characteristics | Unclear | Low | Low | Unclear | Unclear | Unclear | Unclear | Unclear | Unclear | Low | Unclear | Unclear |
| Allocation concealment | Unclear | Low | Unclear | Unclear | Unclear | Unclear | Unclear | Unclear | Unclear | Unclear | Unclear | Unclear |
| Random housing | Unclear | Unclear | Unclear | Unclear | Unclear | Unclear | Unclear | Unclear | Unclear | Unclear | Unclear | Unclear |
| Blinding of researchers | Unclear | Unclear | Unclear | Unclear | Unclear | Unclear | Unclear | Unclear | Unclear | Unclear | Unclear | Unclear |
| Random outcome assessment | Unclear | Unclear | Unclear | Unclear | Unclear | Unclear | Unclear | Unclear | Unclear | Unclear | Unclear | Unclear |
| Blinded outcome assessment | Unclear | Unclear | Unclear | Unclear | Unclear | Unclear | Unclear | Unclear | Unclear | Unclear | Unclear | Unclear |
| Incomplete outcome data | Low | Low | Low | Unclear | Low | Low | Low | Low | Low | Low | Low | Low |
| Selective outcome reporting | Low | Low | Low | Low | Low | Low | Low | Low | Low | Low | Low | Low |
| Other sources of bias | Unclear | Unclear | Unclear | Unclear | Unclear | Low | Unclear | Unclear | Unclear | Low | Unclear | Unclear |

**Overall Conclusion**

In the 12 preclinical publications evaluated, the standard metrics of randomization, allocation concealment, and blinding were consistently not specified or briefly mentioned. Consequently, the risk of bias for these domains was predominantly judged as "Unclear". However, most studies were found to have a "Low" risk of bias concerning incomplete outcome data and selective outcome reporting, as there were no indications of unexplained animal exclusions or unreported outcomes. Additionally, no publication explicitly specified robust randomization or blinding procedures, making it difficult to rule out potential bias. Overall, while no major methodological flaws were apparent, future studies would benefit greatly from more systematic reporting on these key aspects to strengthen the validity of their preclinical findings.

**Risk of Bias Analysis (QUADAS-2 tool)**

The methodological quality of the 2 clinical trials was assessed using the QUADAS-2 tool, an adaptation of the Cochrane RoB tool for animal studies. This tool covers four domains:

- Patient selection
- Index test
- Reference standard
- Flow and timing

Each point is rated:

- low risk
- unclear risk
- high risk

**Li et al. (2023)**

Title: Immuno‑PET of colorectal cancer with a CEA‑targeted [^68^Ga]Ga‑nanobody: from bench to bedside.

- **Clinical settings:** A phase I trial in nine patients with primary or metastatic colorectal cancer.
- **Assessment:** The process of patient selection and the blinding of the index test or reference standard interpreters were not specified, while the flow and timing for confirming lesions were also not fully detailed → "Unclear" risk across all key domains.

**Huang et al. (2025)**

Title: CD38-specific immunoPET imaging for multiple myeloma diagnosis and therapeutic monitoring: preclinical and first-in-human studies.

- **Clinical settings:** A first-in-human trial in two patients with multiple myeloma.
- **Assessment:** Details on patient selection and blinding were not provided, resulting in an "Unclear" risk for those domains. The flow and timing of the comparative scans were well-described and consistent → "Low" risk for that domain.

**Risk of Bias Table (QUADAS-2)**

| **QUADAS-2 domain** | **Li et al. (2023)** | **Huang et al. (2025)** |
| --- | --- | --- |
| Patient selection | Unclear | Unclear |
| Index test | Unclear | Unclear |
| Reference standard | Unclear | Unclear |
| Flow and timing | Unclear | Low |

**Overall Conclusion**

For the two first-in-human clinical trials, the risk of bias was predominantly judged as "Unclear" due to a lack of detailed information on patient selection and blinding procedures. However, the flow and timing of the comparative scans were generally well-described, with one study rated at "Low" risk for this domain. Overall, a comprehensive assessment of bias was limited by the minimal methodological detail provided, which is a common feature of early-phase clinical studies.
